# Polyolefin-Based Smart Self-Healing Composite Coatings Modified with Calcium Carbonate and Sodium Alginate

**DOI:** 10.3390/polym16050636

**Published:** 2024-02-27

**Authors:** Muddasir Nawaz, Rana Abdul Shakoor, Noora Al-Qahtani, Jolly Bhadra, Noora Jabor Al-Thani, Ramazan Kahraman

**Affiliations:** 1 Center for Advanced Materials (CAM), Qatar University, Doha 2713, Qatar; m.nawaz@qu.edu.qa (M.N.); noora.alqahtani@qu.edu.qa (N.A.-Q.); 2 Department of Mechanical and Industrial Engineering, Qatar University, Doha 2713, Qatar; 3 Centeral Laboratories Unit, Qatar University, Doha 2713, Qatar; 4 Qatar University Young Scientist Center, Qatar University, Doha 2713, Qatar; jollybhadra@qu.edu.qa (J.B.); n.al-thani@qu.edu.qa (N.J.A.-T.); 5 Department of Chemical Engineering, Qatar University, Doha 2713, Qatar; ramazank@qu.edu.qa

**Keywords:** anti-corrosive pigment, calcium carbonate, polyolefin coating, sodium alginate, corrosion inhibition, self-healing

## Abstract

Corrosion-related damage incurs significant capital costs in many industries. In this study, an anti-corrosive pigment was synthesized by modifying calcium carbonate with sodium alginate (SA), and smart self-healing coatings were synthesized by reinforcing the anti-corrosive pigments into a polyolefin matrix. Structural changes during the synthesis of the anti-corrosive pigment were examined using scanning electron microscopy (SEM) and X-ray diffraction (XRD) analysis. Moreover, thermal gravimetric analysis confirmed the loading of the corrosion inhibitor, and electrochemical impedance spectroscopic analysis revealed a stable impedance value, confirming the improved corrosion resistance of the modified polyolefin coatings. The incorporation of the anticorrosive pigment into a polyolefin matrix resulted in improved pore resistance properties and capacitive behavior, indicating a good barrier property of the modified coatings. The formation of a protective film on the steel substrate reflected the adsorption of the corrosion inhibitor (SA) on the steel substrate, which further contributed to enhancing the corrosion resistance of the modified coatings. Moreover, the formation of the protective film was also analyzed by profilometry and elemental mapping analysis.

## 1. Introduction

The phenomena associated with corrosion-induced degradation are not only complex but also lead to substantial economic losses, resulting in significant operational and maintenance expenses. Corrosion failures pose critical challenges, bringing the substantial risk of premature metal deterioration. The associated maintenance and additional costs incurred due to the replacement of corroded components result in a significantly higher economic burden. The corrosion of metals is mostly due to the presence of water and oxygen. The complete removal of these elements is difficult; however, effective prevention can be achieved by isolating the metal from the external environment through various protective coatings. In response to these challenges, polymeric coatings have emerged as a practical and promising solution for corrosion prevention in a multitude of applications. A dense and durable polymeric coating offers valuable passive corrosion protection by insulating the metal from the corrosive environment [1]. In addition, active corrosion protection can be provided by adding anti-corrosive pigments into polymeric coatings, which helps to retard the corrosion activity [2,3,4]. Anti-corrosive pigments are formulated by loading corrosion inhibitors and self-healing agents into nanocarriers, which are sensitive to external stimuli, i.e., pH, mechanical load, and temp [5,6]. These nanocarriers play a vital role in preventing any unwanted chemical reaction between the polymer matrix and the loaded active species. Furthermore, they serve as a protective shield, effectively preventing any undesirable leaching effects [7]. Additionally, the incorporation of nanocarriers also serves a dual purpose as a filler material that reduces the pores within the coatings, which eventually makes the coating denser and enhances its overall barrier properties.

Polymeric coatings have gained widespread recognition as a reliable solution for corrosion prevention [8,9]; among their diverse range, polyolefin coatings have been studied less. Polyolefin coatings have been widely used for corrosion prevention, owing to their hydrophobic nature. They form a dense layer, which prevents water penetration and provides excellent adhesion to the substrate. In addition to polyolefin coatings providing excellent corrosion resistance, they are also environmentally friendly and UV resistant [10]. However, one of the main challenges associated with polyolefin coatings is their weak inhibition of corrosion propagation at the metal interface. This issue is primarily due to the highly susceptible nature of polyolefin matrices, which can be further compounded by the addition of anti-corrosion pigments. Incompatibility issues arising from the interactions between these pigments and the polyolefin network can lead to the formation of defects at the pigment–polymer interface, thereby further increasing the susceptibility to corrosion [11]. Furthermore, in contrast to conventional epoxy primers, which can be loaded heavily with pigments, the task of achieving a high inhibitor load within polyolefin coatings can be challenging and is not always feasible. Consequently, the absence of corrosion inhibitors within the coating formulation leads to ineffective corrosion progress stoppage, resulting in significant material loss [12,13]. This outcome is frequently associated with the absence of corrosion inhibitors, leading to ineffective corrosion control and significant loss of the bare material [14,15]. Moreover, the initiation of corrosion processes can cause the formation of strong pH gradients at the steel interface and on coated steel, with pH gradients exceeding 6–7 units between the anodic and cathodic zones. Although polyolefin chemistry is rather stable under pH changes, such large gradients often induce de-adhesion due to cathodic delamination or anodic undermining and, ultimately, due to coating failure, with severe damage to both the coating and the bare materials.

To address these challenges, researchers have explored the use of various materials to enhance the corrosion inhibition properties of polyolefin coatings. One such material is alginate, which can form a physical gel that helps to create a protective film on metal surfaces. Alginates have been readily used as a corrosion inhibitor due to their formation of a physical gel that helps to form a protective film on metal surfaces [16]. Among them, sodium alginate has been widely used for the corrosion inhibition of different metals in various environments [17,18]. I.B. Obot et al. used sodium alginate (SA) for the corrosion protection of API X60 carbon steel in a saline medium [19]. A. Jmiai et al. used SA as a corrosion inhibitor for copper in a hydrochloric acid environment [20]. However, to the best of our knowledge, there is no study about the use of SA as an anti-corrosive pigment with CaCO_3_ that was further incorporated into a polyolefin matrix for the corrosion protection of steel. Considering the market potential for polyolefin coatings reinforced with anti-corrosion pigments, it is essential to highlight the limited research on this topic and the need for novel approaches. The present study aimed to develop a new anti-corrosion pigment by modifying CaCO_3_ to load a corrosion inhibitor. The modified CaCO_3_ was then incorporated into a polyolefin matrix for the formulation of a protective coating for the protection of a steel substrate. It is important to emphasize the paucity of research on polyolefin coatings reinforced with anti-corrosion pigments and the limited discussion of available alternatives. Aiming to develop novel anti-corrosion pigments, this work specifically explores the potential for the corrosion inhibition properties of the coating. The addition of CaCO_3_/Alg provides better protection against corrosion and prevents electrolyte uptake.

## 2. Materials and Methods

### 2.1. Materials

Calcium carbonate (CaCO_3_), sodium salt alginic acid (NaC_6_H_7_O_6_), sodium chloride (NaCl), and absolute ethanol were purchased from Sigma-Aldrich. Polyolefin dispersion (CANVERA™ 1110) was purchased from Dow Chemicals. Plain carbon steel coupons (35 mm × 35 mm × 1.0 mm) were provided by a local supplier with the same composition as in our previous research [8].

### 2.2. Development of Anti-Corrosive Pigment

**Figure 1:**
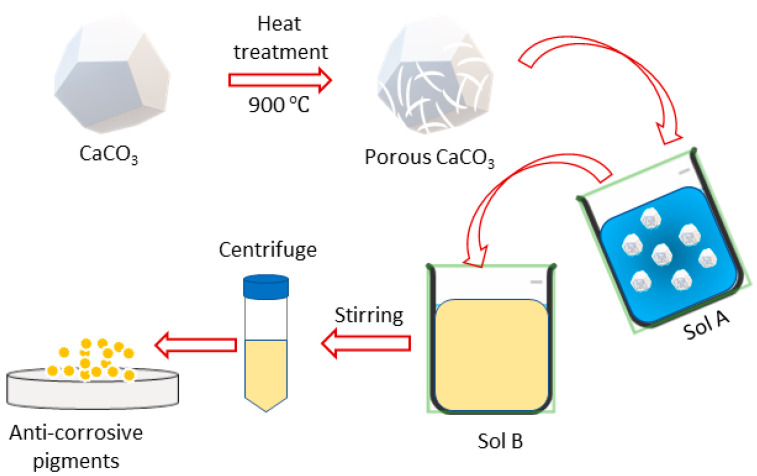
Schematic diagram of synthesis of anti-corrosive pigment.

The formation of a porous calcium carbonate (CaCO_3_) structure was a mandatory step in our study so that it could hold the corrosion inhibitor within its porous structure. To achieve this, the CaCO_3_ was heated at 900 °C for 3 h in the furnace to obtain a porous structure. The carburizing effects converted the CaCO_3_ into calcium oxide (CaO) and carbon dioxide (CO_2_), and the removal of CO_2_ converted the pores into CaO (Equation (1)). The porous CaO obtained after the heat treatment was dispersed in deionized water, at 3 wt.% relative to the deionized (D.I) water, labeled as solution A (Equation (2)). The calcium oxide, when introduced into the aqueous environment, underwent yet another transformative phase transition, converting into calcium hydroxide (Ca(OH)_2_) (Figure 1).

(1) $$CaCO_{3}\to CaO+CO_{2}$$

(2) $$CaO+H_{2}O\to Ca(OH{)}_{2}$$

Solution B was prepared by adding 5 mg/mL of sodium salt alginic acid to D.I water. Once the sodium alginic acid completely dissolved and became a homogeneous mixture, solution A was added dropwise to solution B, with moderate stirring at room temperature. The solution was stirred overnight, followed by centrifugation and overnight drying at 60 °C to obtain a dried anti-corrosive pigment product (CaCO_3_/Alg).

### 2.3. Coating Formulation and Application to Steel Substate

The surface preparation of a substrate is important before a coating application. The steel substrates were polished with 120 grit size emery papers to make a rough surface, which helps to provide good adhesion strength due to mechanical interlocking. The steel substrate was thoroughly rinsed with distilled water and ethanol before the coating application to make sure the surface was free from any contamination. Then, 1 wt.% of the anti-corrosive pigment was used as an additive for the polyolefin coating. The anti-corrosive pigment was initially dispersed into deionized water for better dispersion, which was later added to the polyolefin solution. Continuous stirring was carried out to obtain a homogeneous mixture before applying the coating with a dip-coating technique. The polyolefin coatings were cured by heating in an oven at 160 ± 5 °C for 15 min. Two types of coatings, including a non-modified (blank polyolefin) coating, which did not have the additive, and a modified coating, which contained the anti-corrosive pigment (CaCO_3_/Alg), were formulated for clear comparison purposes. The thickness of the dried polyolefin coatings was kept at 50 ± 5 µm for a clear comparison.

### 2.4. Characterization

Morphological analysis of the synthesized anti-corrosive pigments was carried out using scanning electron microscopy (SEM). The phase purity of the CaCO_3_/and CaCO_3_/Alg was examined by X-ray diffraction (XRD). Frontier transform infrared spectroscopy (FTIR) was performed to determine any structural changes and the chemical composition. Thermal gravimetric analysis (TGA) and Brunauer–Emmett–Teller (BET) analysis were used to determine the loading capability. The coating thickness of the coated samples was measured by a gauge meter (PosiTector 6000, DeFelsko, Ogdensburg, NY, USA). The corrosion inhibition properties of the coated samples were evaluated by electrochemical impedance spectroscopy (EIS) in a 3.5 wt.% NaCl solution. The detailed specifications of all the techniques are mentioned in our previous work [21].

## 3. Results and Discussion

### 3.1. Structural Analysis

**Figure 2:**
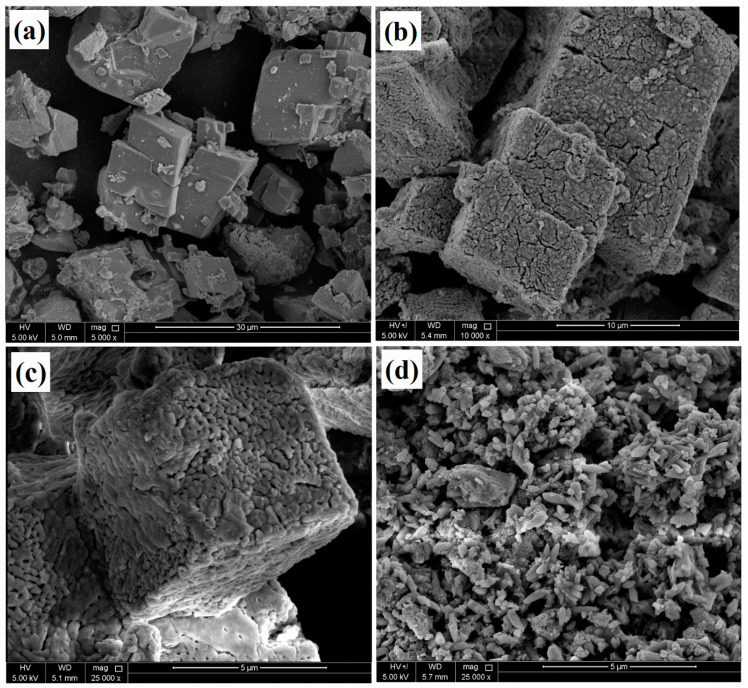
SEM images of the CaCO_3_ (**a**) before and (**b**,**c**) after heat treatment, (**d**) CaCO_3_/Alg.

SEM images of the CaCO_3_ before and after the heat treatment and loading with the corrosion inhibitor are depicted in Figure 2, showing the structural changes during the heat treatment and loading. Figure 2a shows a smooth surface with a hexagonal geometry of the CaCO_3_, which converted into a porous structure after the heat treatment of the calcium carbonate, as shown in Figure 2b,c. However, the heat treatment did not affect the hexagonal structure of the CaCO_3_, which remained the same, but some cracks and porosity were observed due to the emission of CO_2_ during the heat treatment. Figure 2d depicts the anti-corrosive pigment that was loaded into CaCO_3_ with sodium alginate. The particle size was reduced significantly after loading with the inhibitor, and the calcium carbonate deformed from its original loading process due to the treatment with water. The sodium alginate flakes filled the pores and covered the CaCO_3_, which was also observed during the BET analysis.

**Figure 3:**
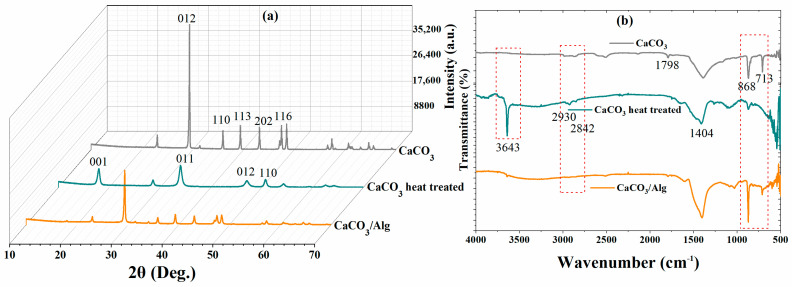
(**a**) XRD and (**b**) FTIR analysis of CaCO_3_ (Grey color), CaCO_3_ after heat treatment (Green color), and CaCO_3_/Alg (Orange color).

XRD analysis of the CaCO_3_ before and after the heat treatment was carried out to evaluate the changes in the structure. A hexagonal crystal structure was observed in the XRD pattern, corresponding to the CaCO_3_ [22]. Moreover, peaks were observed at (012), (110), (113), (202), and (116) for the CaCO_3_, matching the “ICDD 98-019-1852”, and they are presented in Figure 3a. Moreover, it is noteworthy that the new patterns observed due to changes in the crystalline structure (face-centered cubic), due to the porosity after the heat treatment, correspond to the ICDD 98-020-2228 [23]. The XRD pattern of the anti-corrosive pigment (CaCO_3_/Alg) exhibited almost identical peaks to the calcium carbonate before the heat treatment. However, the intensity of the peaks was reduced due to the presence of sodium alginate in the calcium carbonate.

The FTIR spectra of calcium carbonate, heat-treated calcium carbonate, and calcium carbonate loaded with SA are depicted in Figure 3b. A low-intensity band was observed for the heat-treated CaCO_3_ at 3643 cm^−1^, which corresponds to the O-H bond. Moreover, the minor peaks at 2930 and 2842 cm^−1^ correspond to C=O due to the carbonate ion, as shown in Figure 3b. The strong peak at 1404 cm^−1^ corresponds to the C-O bond. The Ca-O bond can also be seen at 713 cm^−1^. The strong O-H bond observed at 3643 cm^−1^ was due to the remaining hydroxide after the heat treatment [24]. Like CaCO_3_, a C-O bond at 1404 cm^−1^ was also observed after the heat treatment. A relatively low-intensity peak can also be observed at 868 cm^−1^, which presents the C-O bond. Furthermore, the O-H functional group was observed between 3200 and 3400 cm^−1^ due to the presence of sodium alginate in the anti-corrosive pigment (CaCO_3_/Alg) [25]. Symmetric and asymmetric vibrations were observed at 1406 and 1648 cm^−1^ due to the presence of carboxylate salt ions [26].

### 3.2. TGA and BET Analysis

**Figure 4:**
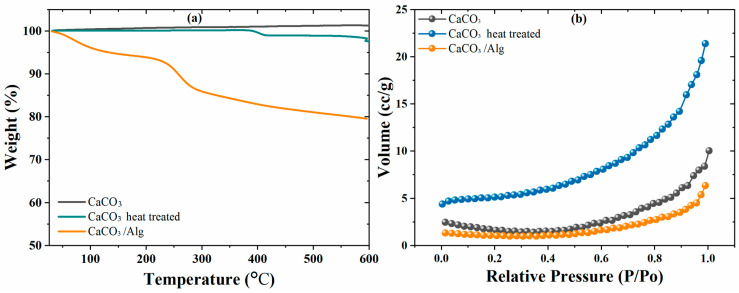
(**a**) TGA and (**b**) BET analysis of CaCO_3_, CaCO_3_ after heat treatment, and CaCO_3_/Alg.

Thermal gravimetric gravitation analysis (TGA) was performed from room temperature to 600 °C to evaluate the thermal stability and weight loss characteristics of both the CaCO_3_ and CaCO_3_/Alg. Figure 4a shows no weight loss for the CaCO_3_; however, almost 2 wt.% weight loss was observed after the heat treatment of the CaCO_3_ due to the removal of any residual CO_2_. Furthermore, the CaCO_3_ encapsulated with alginic acid had a total weight loss of ~20 wt.%. The initial weight loss at 100 °C was due to the presence of moisture content, while the weight loss observed between 200 and 300 °C was due to the thermal degradation of sodium alginate. The 15 wt.% weight loss represents the loading capability of the corrosion inhibitor (SA) in the CaCO_3_.

Moreover, the changes in the surface area and porous nature of the CaCO_3_ before and after the heat treatment and loading with alginic acid were evaluated by BET analysis. Figure 4b shows that the specific surface area of the CaCO_3_ was 4.73 m^2^/g, with a pore volume of 0.0156 cc/g. After the heat treatment, when the CaCO_3_ decomposed, the porosity increased, which led to a higher surface of 16.24 m^2^/g, and a 0.0332 cc/g pore volume was obtained. Furthermore, the specific surface area of the anticorrosive pigment (CaCO_3_/Alg) significantly decreased to 3.145 m^2^/g. Corresponding to the specific surface area, the pore volume was also reduced to 0.0098 cc/g due to the effective filling of the corrosion inhibitor in the CaCO_3_. The results of the BET analysis also confirm the successful and effective loading, as observed in TGA results.

### 3.3. Electrochemical Analysis

**Figure 5:**
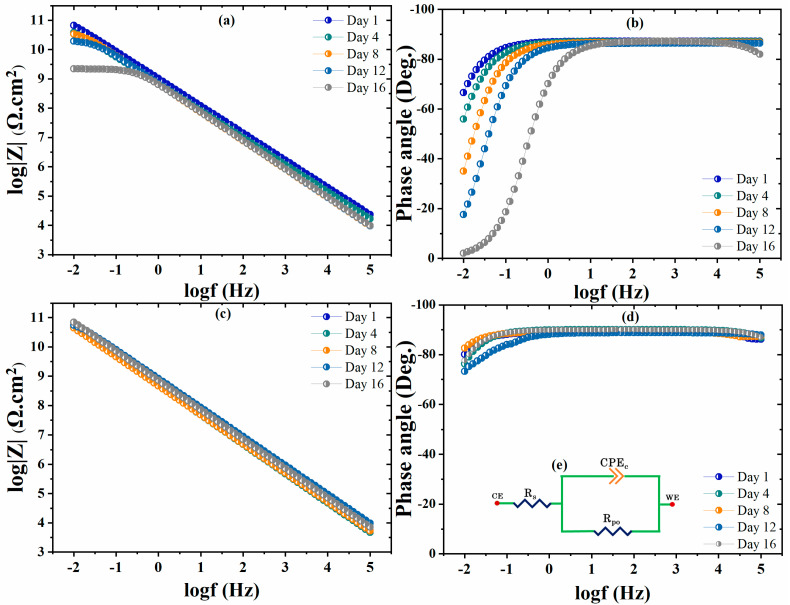
The EIS Bode plots of the (**a**,**b**) non-modified and (**c**,**d**) modified coating.

The corrosion inhibition behavior of the non-modified and modified coated samples was analyzed by EIS analysis after 16 days of immersion in 3.5 wt.% NaCl solution. The fitted EIS spectra for the non-modified coatings (blank polyolefin) and modified coatings (CaCO_3_/Alg) are depicted in Figure 5. Furthermore, the equivalent circuit used to obtain the fitting data is depicted in Figure 5d. Initially, the EIS spectra for the non-modified coating showed a higher impedance value of ~100 G Ω·cm^2^ for one week, which declined to 1 G Ω·cm^2^ on the 16th day of immersion. The electrolyte uptake through the micropores caused a decrease in the overall corrosion protective behavior of the non-modified polyolefin coating. The broadening of the horizontal area at a lower frequency of the EIS spectra of the non-modified coating shows a decrease in the capacitive behavior of the unmodified coatings. Corrosion activity at the low-frequency region of the phase angle can also be observed due to a shift in the phase angle towards zero (0°). An increase in the active surface area is indicated by a reduction in the capacitive response, which caused a loss in the corrosion protection behavior of the coatings. Overall, it was decreased by two orders of magnitude in the non-modified coating after 16 days of immersion.

Moreover, in contrast, the modified coating showed a stable impedance spectrum over time of approximately 100 G Ω·cm^2^ due to the reduction in the micropore area of the coating. The loaded anti-corrosive pigment (CaCO_3_/Alg) also acted as filler material in the modified coating, making the coating denser and reducing the active surface area of the coating [21]. The effective filling and corrosion inhibition due to the anti-corrosive pigments prevented electrolyte uptake and maintained its capacitive behavior. The EIS spectra of the modified coating are depicted in Figure 5c, showing that the capacitive region remained stable and that there was no resistive region observed, unlike in the non-modified coating. Moreover, the phase angle values of the modified coatings remained stable, around −90°, at the higher and normal frequency regions, while they were −80° at the low-frequency region, showing the better capacitive behavior of the modified coatings [27]. The better corrosion protection of the modified coating could also be due to the presence of the anti-corrosive pigment. It is reported that the corrosion inhibitor (alginic acid) can be adsorbed on steel substrate, which reduces the active area and isolates the steel substrate from water/electrolytes, resulting in a reduction of corrosion activity [19]. The corrosion inhibition and self-healing properties of the modified coating are evaluated and discussed in Section 3.5.

**Figure 6:**
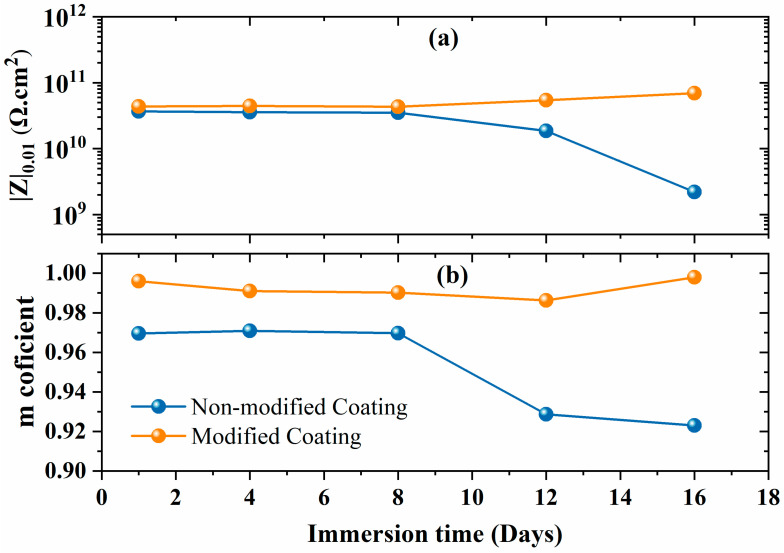
(**a**) Low impedance values with (**b**) m-coefficient of capacitance of non-modified and modified coatings.

The low-frequency impedance spectra (|Z|_0.01_) of the non-modified and modified coating are depicted in Figure 6a. The non-modified coating shows a decrease in the low-frequency impedance spectra, dropping below 10 G Ω·cm^2^ after 10 days of immersion. In contrast, the modified coating shows a stable value above 10 G Ω·cm^2^ without any reduction at low frequencies. This suggests that the coating barrier properties of the modified coating improved due to the prevention of electrolyte uptake compared to the non-modified coating. The m-coefficient factor defines the capacitive or resistive behavior of the coatings; for a pure capacitive, its value is −1, and for a pure resistor, its value is 0. The m-coefficient factor values remained around ~1 for the modified coating, which indicates that the coating behaved like a pure capacitive (Figure 6b). Moreover, the results are also consistent with the EIS Bode spectra, where the phase angle was close to −90° over a wide frequency range. The EIS results also suggest that the CaCO_3_/Alg additives had compatibility with the polyolefin matrix. However, the m-coefficient value of the non-modified coating decreased to 0.92, which is less compared to that of the modified coatings. This observed decline was due to the formation of a resistive region after approximately one day of immersion. Overall, both coatings showed capacitive behavior.

**Figure 7:**
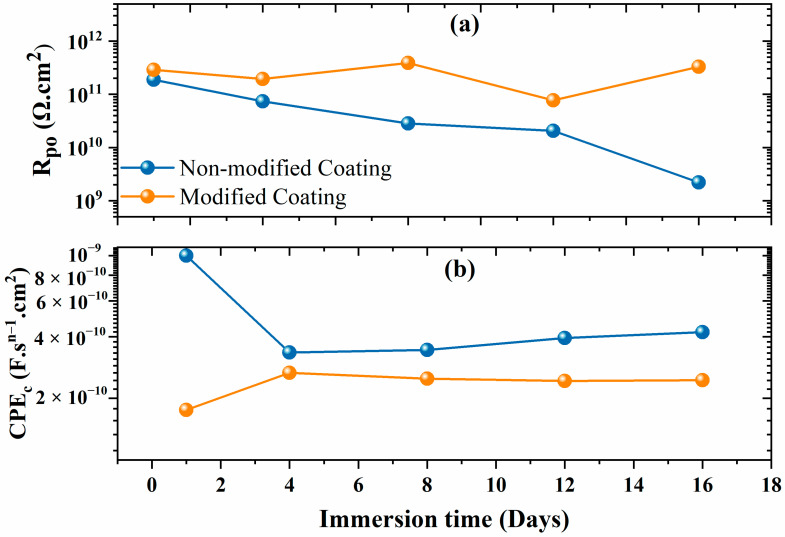
(**a**) Pore resistance and (**b**) CPE_c_ of the non-modified and modified coatings.

Figure 7 displays the fitting parameters derived from the numerical modeling of the EIS data. The non-modified and modified coatings show a pore resistance (R_po_) value near 100 G Ω.cm^2^ at the start of the immersion time. The gradual reduction in the R_po_ value over time for the non-modified coating can be attributed to the electrolyte absorption facilitated by the porous nature of polyolefin (Figure 7a). This phenomenon is further confirmed by the diminishing phase angle and the emergence of a more distinct resistive plateau at lower frequencies. [28]. The R_po_ value decreased by two orders of magnitude for the non-modified coatings, highlighting the lower degree of protection that the coating provided and indicating coating deterioration. However, the R_po_ obtained for the modified coatings was slightly higher at the initial immersion and it remained above 100 G Ω·cm^2^ even after 16 days of immersion. This justifies the improved barrier performance due to the anti-corrosive pigment (CaCO_3_/Alg) and demonstrates that the pore resistance of the coating was greatly increased by this system. The phase angle was stable at incredibly low frequencies, illuminating the greatly enhanced and durable barrier properties. The lower CPE_c_ value of the modified coatings compared to that of the non-modified coatings elucidates the better corrosion protection of the steel. Overall, the modified coatings exhibited lower and more consistent CPE_c_ admittance values compared to those of the non-modified coatings. This observation serves as compelling evidence of their heightened resistance to electrolyte infiltration and underscores their enhanced corrosion protection potential.

### 3.4. Contact Angle

**Figure 8:**
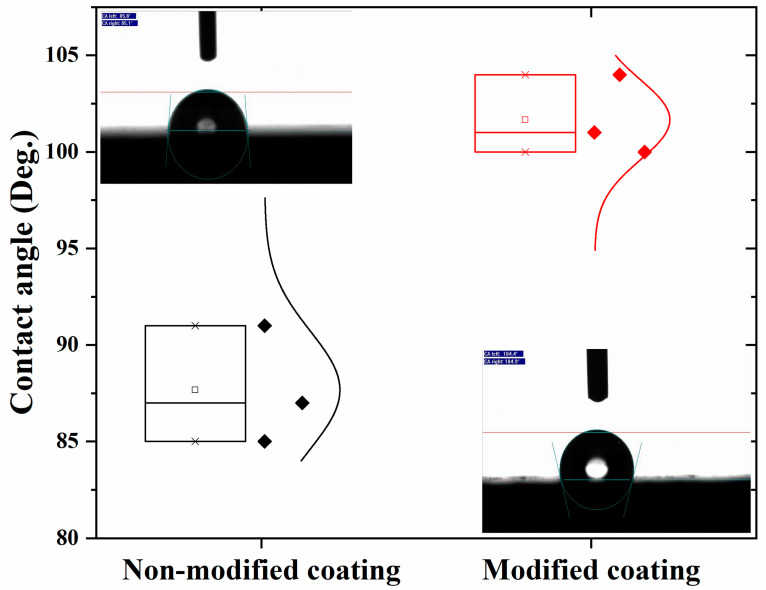
Contact angle measurement for (**a**) non-modified and (**b**) modified coating.

Electrochemical analysis showed a capacitive behavior and that the modified coating prevented water uptake. Then, the hydrophobic nature of the coated samples was further evaluated by the contact angle measurement. The sessile drop method was used for the measurement of the contact angle. Both coatings showed a hydrophobic nature due to their polyolefin coating. Figure 8 depicts that the non-modified coating had a maximum contact angle of around 90°; in contrast, the contact angle of the modified coatings was higher than 100°. The addition of the anti-corrosive pigments made the coating denser, enhancing the contact angle value. These results are also consistent with the EIS results because a higher contact angle causes a higher m-coefficient value with a capacitive behavior response.

### 3.5. Self-Healing Mechanism

**Figure 9:**
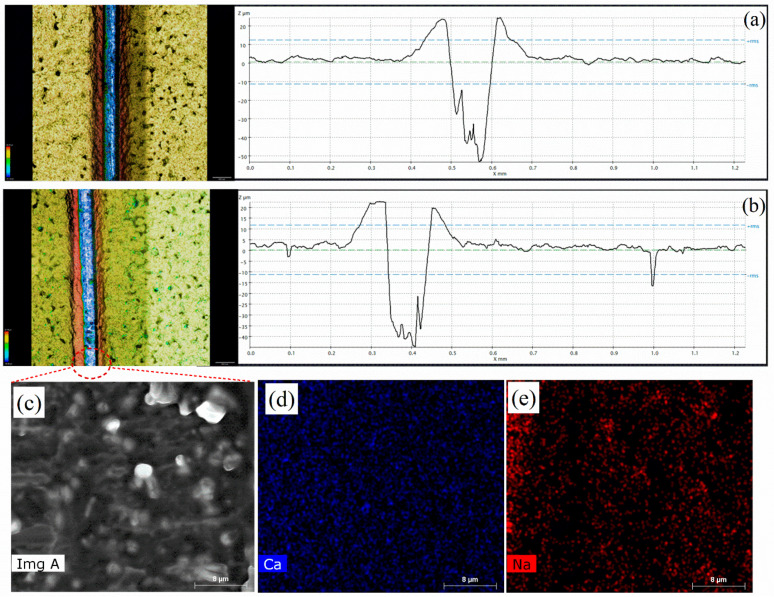
Profilometer images of the (**a**) non-modified and (**b**) modified coatings; (**c**) SEM and (**d**,**e**) elemental mapping image of scratched region of modified coatings.

The corrosion inhibition effect due to self-healing (protective film) was studied for the non-modified and modified coatings by making a scratch on the coating. The coated samples, both modified and non-modified, were scratched to evaluate their self-healing abilities. The scratched samples were immersed in the same environment (3.5 wt.% NaCl) as used for the EIS analysis. The anticorrosive pigment formed a protective layer on the steel substrate when the coating was damaged. Both the modified and non-modified scratched samples were subjected to scanning under profilometry to evaluate the formation of a protective film by analyzing the varying depths over the scratch area. Figure 9a shows a profilometer image of the scratched non-modified coatings, which did not show any change in the depth compared to the original depth, which was 50 µm. However, the modified scratched sample in Figure 9b shows a significant drop in the scratch depth area to 40 µm compared to the initial depth of 50 µm. This decrease of 10 µm in the coating depth occurred due to the formation of a protective film on the steel substrate, which filled the gap and reduced the depth. Sodium alginate has a carboxylate group, COONa, which has a high occupied molecular orbital as its backbone for the formation of a protective film on steel substrate. The unoccupied d-orbital of iron interacts with active sites of sodium alginate available at (-COONa) and develops a protective film, which eventually decreases the scratch depth and helps in the corrosion inhibition of the coating [29]. The SEM results, along with the elemental mapping of the scratched area of the modified coatings, are depicted in Figure 9c–e. Figure 9c shows a slightly irregular surface, which formed due to the protective film on the steel substrate, and the elemental mapping depicted in Figure 9d,e confirms that the protective layer formation was due to the anti-corrosive pigment (CaCO_3_/Alg). The presence of sodium and calcium in the scratched area further confirms that the protective film was formed due to the adsorption of the anti-corrosive pigment on the steel substrate.

**Figure 10:**
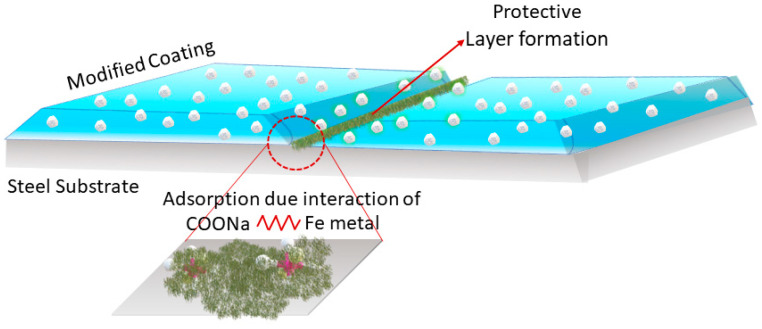
Schematic diagram of the self-healing mechanism of the modified coating.

Figure 10 shows a schematic diagram of the protective film formation in the modified coating. The scratched area formed a protective layer due to the adsorption of the protective layer on the steel substrate. The sodium alginates had the ability to form a protective film on the steel substrate due to the interaction between the functional group (-COONa) of sodium alginate and the metal ions. The film led to a decrease in the depth of the scratch of the coatings, as demonstrated in the profilometry image of the modified coating. The film also helped to isolate the electrolyte from the steel substrate, due to which the electrolyte uptake was less in the modified coatings [30].

## 4. Conclusions

The results of the comprehensive analysis and experimentation confirm the successful loading of 15 wt.% of a corrosion inhibitor into calcium carbonate, as validated by TGA analysis. Meanwhile, a significant decrease in the specific surface area from 16.24 m^2^/g to 3.145 m^2^/g was also observed due to the effective filling of the CaCO_3_ pores during the BET analysis. The incorporation of calcium carbonate particles loaded with sodium alginate into polyolefin coatings showed significant improvements in the corrosion inhibition efficiency of the coatings. The stable impedance value at a lower-frequency region, determined with electrochemical impedance spectroscopy, indicates the good barrier property of the modified coatings. The EIS parameter values of the modified coatings exhibited improved pore resistance and capacitive behavior, which suggest the effective filling of the anti-corrosive pigments and the formation of a protective film by the adsorption of SA on the steel substrate. These eventually contributed to the coatings’ improved barrier properties. This protective film formation was due to the interaction between the functional group (-COONa) of sodium alginate and the metal ions, which led to the development of a stable and effective protective film. The contact angle increment from 90° to 100° shows the hydrophobic nature of the coating. The self-healing ability of the damaged modified coating was observed by profilometry scanning, where a decrease of 10 µm in the depth was observed due to the formation of the protective film. The outcomes of this study suggest that modified polyolefin coatings have significant potential for use in various industrial applications to protect against corrosion-related damages.

## Data Availability

The data are currently unavailable due to being part of ongoing research.
